# Skin Autofluorescence as a Potential Adjunctive Marker for Cardiovascular Risk Assessment in Type 2 Diabetes: A Systematic Review

**DOI:** 10.3390/ijms25073889

**Published:** 2024-03-31

**Authors:** Delia Reurean-Pintilei, Anca Pantea Stoian, Claudia-Gabriela Potcovaru, Teodor Salmen, Delia Cinteză, Roxana-Adriana Stoica, Sandra Lazăr, Bogdan Timar

**Affiliations:** 1 Doctoral School of Medicine and Pharmacy, “Victor Babes” University of Medicine and Pharmacy, 300041 Timisoara, Romania; 2 Centre for Molecular Research in Nephrology and Vascular Disease, “Victor Babes” University of Medicine and Pharmacy, 300041 Timisoara, Romania; 3 Department of Diabetes, Nutrition and Metabolic Diseases, Consultmed Medical Centre, 700544 Iasi, Romania; 4 Diabetes, Nutrition and Metabolic Diseases Department, “Carol Davila” University of Medicine and Pharmacy, 050474 Bucharest, Romania; 5 9th Department of Physical Medicine and Rehabilitation, “Carol Davila” University of Medicine and Pharmacy, 050474 Bucharest, Romania; 6 Doctoral School, “Carol Davila” University of Medicine and Pharmacy, 050474 Bucharest, Romania; 7 First Department of Internal Medicine, “Victor Babes” University of Medicine and Pharmacy, 300041 Timisoara, Romania; 8 Department of Hematology, Emergency Municipal Hospital Timisoara, 300041 Timisoara, Romania; 9 Second Department of Internal Medicine, “Victor Babes” University of Medicine and Pharmacy, 300041 Timisoara, Romania; 10 Department of Diabetes, Nutrition and Metabolic Diseases, “Pius Brinzeu” Emergency Hospital, 300723 Timisoara, Romania

**Keywords:** AGE, SAF, T2DM, CV risk factors

## Abstract

Diabetes mellitus (DM), due to its long-term hyperglycemia, leads to the accumulation of advanced glycation end-products (AGEs), especially in the vessel walls. Skin autofluorescence (SAF) is a non-invasive tool that measures AGEs. DM patients have a rich dietary source in AGEs, associated with high oxidative stress and long-term inflammation. AGEs represent a cardiovascular (CV) risk factor, and they are linked with CV events. Our objective was to assess whether SAF predicts future CV events (CVE) by examining its association with other CV risk factors in patients with type 2 DM (T2DM). Additionally, we assessed the strengths and limitations of SAF as a predictive tool for CVE. Following the Preferred Reporting Items for Systematic Reviews and Meta-Analyses methodology, we conducted a systematic review with CRD42024507397 protocol, focused on AGEs, T2DM, SAF, and CV risk. We identified seven studies from 2014 to 2024 that predominantly used the AGE Reader Diagnostic Optic tool. The collective number of patients involved is 8934, with an average age of 63. So, SAF is a valuable, non-invasive marker for evaluating CV risk in T2DM patients. It stands out as a CV risk factor associated independently with CVE. SAF levels are influenced by prolonged hyperglycemia, lifestyle, aging, and other chronic diseases such as depression, and it can be used as a predictive tool for CVE.

## 1. Introduction

Half of all deaths in people with type 2 diabetes mellitus (T2DM) are caused by cardiovascular disease (CVD), which is common in this population. Even though smoking, dyslipidemia, and high blood pressure (HBP) are traditional risk factors that contribute significantly to the cardiovascular events (CVEs) that occur in these patients, the risk assessment instruments that are currently in use do not adequately take into consideration the risk. This may have been an oversight due to the distinct risks associated with microangiopathy and chronically elevated blood sugar [1]. Advanced glycation end-products (AGEs), which are produced because of hyperglycemia, significantly contribute to the vascular complications associated with diabetes mellitus (DM). However, the levels of AGEs in the serum do not consistently predict the occurrence of subsequent CVE in individuals with T2DM [2].

CVDs claimed the lives of approximately 17.9 million individuals in 2019, comprising nearly a third of the total global deaths. A striking 85% of these fatalities were caused by myocardial infarctions (MI) and strokes. Ischemic heart disease (IHD) is a significant global cause of mortality, accounting for 16% of all fatalities, as reported by the World Health Organization. From 2000 to 2019, its incidence has steadily risen. According to the most recent report from 2023 by the World Heart Federation, IHD remains the leading cause of CV-related deaths globally, constituting four-fifths of CVD deaths. A significant majority of these occur in low- and middle-income countries, with the highest rates reported in Central Europe, Eastern Europe, and Central Asia regions. Notably, up to 80% of premature MI and strokes can be prevented with proper prevention and management. Stroke is considered the second leading cause of death globally, while in low-income countries, it is the third most common cause of death; however, in middle-income, upper-middle-income, and high-income countries, it takes precedence as the leading cause of death [3]. The report underscores the significance of understanding differing risk factors across regions, underscoring the importance for countries to be aware of their unique risk profiles [4,5,6,7].

DM has emerged as a significant global health issue, characterized by a distressing increase in the number of new cases worldwide. Severe complications, including an increased risk of CVD, often accompany this chronic condition. T2DM poses a significant threat to vascular health, which can lead to an increased risk of premature death due to CVEs [8]. The relationship between T2DM and CVD is reciprocal, and both conditions are typically managed simultaneously [9]. DM and CVD, including heart disease and stroke, are among the noncommunicable chronic diseases, along with mental disorders, cancer, and respiratory illnesses [10]. They collectively contribute to 66.5% of all years lived with disability in low-income and middle-income countries. Long-term care and prevention play a pivotal role in reducing complications, disabilities, and the overall burden associated with these conditions [11].

Hyperglycemia is the defining feature of DM and is closely linked to the formation and intensification of CV complications [12,13]. Long-term exposure to high blood sugar is linked with a series of biochemical events that result in the glycation and oxidation of proteins and lipids, ultimately leading to the accumulation of AGEs [14,15]. This group of compounds has been linked to various chronic diseases, including CVEs in DM patients. These substances are created through non-enzymatic glycation involving sugar compound reduction on proteins and/or lipids [16]. Accumulation of AGEs increases the risk for both DM development and overt CVEs [17]. Receptors for AGEs bind with different ligands involved in atherosclerosis development. They are highly expressed where this process intensifies—implicating these receptors’ activation as part of DM-driven vascular lesion promotion [18,19].

In addition to endogenous AGEs, those originating from food sources have been associated with promoting oxidative stress and inflammation. This established association intertwines obesity, DM, and CV pathologies together [20]. Additionally, researchers noted an association between skin autofluorescence (SAF) and heightened risk for diverse populations encountering CVEs [21,22]. This relationship between vascular injury and AGEs has been widely studied, and AGEs have been identified as potential indicators of CVEs and fatalities [21,23,24,25,26,27,28,29,30,31]. A non-invasive technique for quantifying AGEs known as SAF has emerged as a promising instrument for evaluating CV risk. By measuring the fluorescent properties of AGEs in the skin, SAF provides a snapshot of AGE accumulation in the body. The SAF technique involves using specific wavelengths of light to illuminate the skin and then measuring the fluorescent light, reflecting the level of AGEs present [25,32,33]. The non-invasive nature of this method makes it a convenient and practical tool for routine clinical use, considering the potential implications of incorporating SAF into existing risk stratification algorithms and guidelines for managing CV risk in individuals with T2DM [23,31,34,35,36]. Developing innovative methods for evaluating the severity of DM and categorizing CV risk is crucial to providing patients with personalized and effective treatment plans.

In this study, we aim to comprehensively review the existing literature on the adjunctive marker capabilities of SAF in the context of CV risk assessment. By synthesizing findings from various research studies, we seek to elucidate the strengths and limitations of SAF as a predictive tool for CVEs and mortality. Our objective is to provide insights that inform clinical practice and guide future research endeavors in CV risk assessment and management.

## 2. Methods

A systematic review was performed according to the guidelines and recommendations from the Preferred Reporting Items for Systematic Reviews and Meta-Analysis Checklist (PRISMA). The protocol for this review has been registered with the identifier CRD42024507397.

### 2.1. Research Question and Search Strategy

An electronic search for relevant publications was performed using PubMed and Web of Science library databases from 1 January 2013, to February 2024. The following search strategy was used: “(advanced glycation end products OR AGE) AND (Skin autofluorescence) AND (Type 2 Diabetes OR Diabetes Mellitus type 2 OR T2DM) AND cardiovascular risk”. After this search, 123 articles were found (45 from PubMed and 78 from Web of Science). After applying filters for language (English), publication type (original articles), and date range (2013 to the date of the search), 60 articles remained. We eliminated the two duplicates, and then the remaining 58 articles underwent initial title screening, followed by abstract review by two independent reviewers. After excluding studies focused on populations other than the one of interest, those with outcomes different from the ones of interest, as well as meta-analyses and study protocols, five articles remained for full assessment. Afterward, two more studies were identified by manual search of the reference list of the relevant articles and were added.

The research question was framed using the Population, Intervention, Comparison, and Outcome (PICO) method. The population was represented by patients with T2DM in whom AGEs have been assessed using SAF; intervention was represented by the SAF level and its relationship with CV risk factors and outcome. The outcome was defined by the number and/or presence of CV risk factors and/or established CVD in patients with T2DM who have undergone an assessment of SAF, with effect measured by percentage, confidence interval, odds, or relative risks.

### 2.2. Inclusion Criteria

To be included in this review, studies had to meet the following publication criteria: (i) original full-text articles with cohort and cross-sectional studies; (ii) articles from the last ten years; (iii) articles published in English; (iv) on adult human populations.

### 2.3. Exclusion Criteria

Studies were excluded from the analysis if they were (i) on patients without T2DM, (ii) they lacked the CV outcomes, and (iii) if the study had comments, letters to editors, or reviews.

### 2.4. Selection of Studies

Studies that met the following eligibility criteria were included: (1) included patients with T2DM in whom AGE have been assessed using SAF; (2) SAF level and its relationship with CV risk factors and outcome were reported; (3) provided sufficient information such as the corresponding 95% confidence intervals (CIs) or at least *p*-value. Studies were excluded if they: (1) were a letter to the editor, expert opinions, case reports, meeting abstracts, or reviews; (2) were redundant publications; or (3) needed more precise or complete data.

### 2.5. Data Extraction

Two authors used a self-made data extraction table to individually evaluate and extract the following data for each included literature: the first author and year of publication, geographic region, study period, study design, sample size, the average age of participants, CV risk factors, SAF level, confounding factors adjusted, reported outcomes and risk estimates with their corresponding 95% CIs. Any differences of opinion were settled through discussion or consultation with a third author.

### 2.6. Risk of Bias Assessment

Two reviewers independently assessed the quality of the studies using the Newcastle-Ottawa Scale (NOS) [37], a star rating system that evaluates articles on selection, comparability, and outcome criteria. Research papers rated with at least six stars are considered good quality, as seen in Table 1.

### 2.7. Strategy for Data Synthesis

A narrative synthesis of the findings in the studies centered around the SAF level and its relationship with CV risk factors and outcomes. The studies are anticipated to be heterogeneous (study design, study quality, screening methods described, interventions, and outcomes). Therefore, it is expected that a narrative synthesis will be performed, using text and tables to provide a descriptive summary and explanation of study characteristics and findings.

## 3. Results

**Figure 1:**
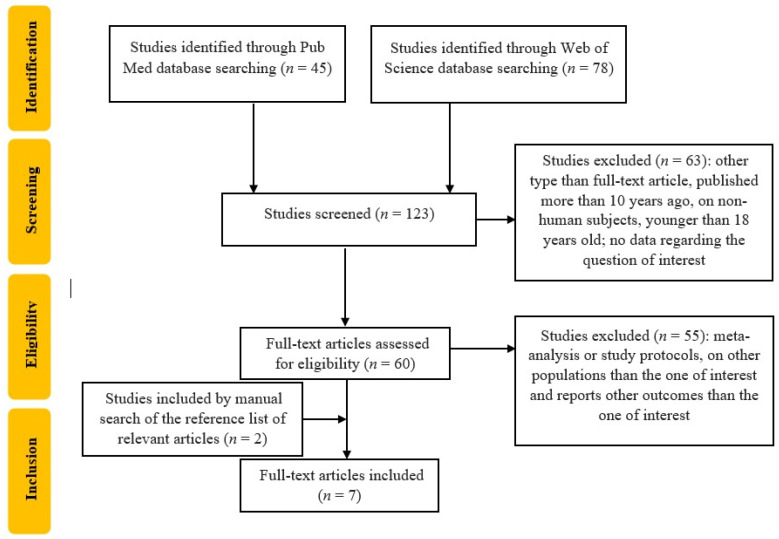
Flowchart of the study selection.

This systematic review incorporates seven studies published between 2014 and 2024 as seen in Figure 1.

Table 2 summarizes some information extracted from the selected studies, as described below. These studies span various global regions, including Europe (France, Spain, The Netherlands) and Asia (Japan, Hong Kong, China), focusing on the relationship between SAF and CVEs in T2DM patients. The duration of the studies is different: from 4 months to 7 years. The average study duration across the reviewed studies is approximately four years and one month. Regarding the study design, one study was retrospective, and six were prospective. The included studies encompassed 8934 patients with an average age of 63 years. The most used SAF tool was AGE Reader Diagnostic Optic (Groningen, The Netherlands); one study used AGE Reader (Selista Inc. Tokyo, Japan), and in one study, the Hefei Institutes of Physical Science, Chinese Academy of Sciences device was used.

## 4. Discussion

We investigated the association between SAF values and CVD and CVEs in patients with T2DM.

The studies reviewed included patients with an average age of 63 years, and the population appeared to be relatively homogeneous, encompassing the transition from adulthood to older age. This age group holds particular physiological relevance, as individuals within this range are at increased risk for CV conditions representing a critical period when the likelihood of CVD escalates significantly, making the study results highly applicable for devising preventive and management strategies in healthcare [45].

The span of four years and one month, as observed across the analyzed studies, represents a significant duration in observing the progression of CV complications in T2DM patients, and in assessing the prognostic utility of SAF in this population. It emphasizes the role of longitudinal research in establishing links and potential causal relationships within the field of DM and CV investigations [38,39,40,41,42,43,44].

The definition of CVEs varies among the studies evaluated. This difference emphasizes the need of specific definitions in harmonizing data across research, as well as the broad and comprehensive character of CVD and related events.

### 4.1. SAF as a Marker for CV Risk Assessment in T2DM Patients

Alkhami et al. [38] and Boersma et al. [42] emphasize the importance of SAF as a marker for CV risk assessment in T2DM patients. The first study focused on a retrospective analysis of 504 uncontrolled or complicated patients who were hospitalized with T2DM, exploring the relationship between SAF and subsequent CVEs, including MI, stroke, revascularization procedures, and CV death over a follow-up of approximately 54 months. Their findings indicate that SAF, measured at admission, significantly predicts later CVEs, even after adjusting for traditional risk factors. Boersma et al. [42] included a broader cohort of 2349 T2DM population, consisting of a mix of newly and previously diagnosed patients from the Lifelines Cohort Study, examining SAF’s predictive value for new CVEs and mortality, with a median follow-up of 3.7 years. Their results show that elevated SAF is strongly associated with the combined outcome of new CVEs or mortality, as well as the incidence of CVD and death as separate outcomes. The study underlines SAF’s superiority in predicting future CVEs and mortality compared to traditional risk factors such as cholesterol or blood pressure levels. While the two separate studies have both demonstrated a connection between elevated SAF levels and an increased likelihood of CVEs and mortality, underscoring the utility of SAF as a non-invasive indicator for identifying individuals at a higher risk for CV complications, Boersma et al. [42] provide a broader perspective by evaluating SAF’s predictive capacity in a larger, more diverse cohort with a more extended follow-up period. One important aspect to be taken into account is the adjustments in analysis: Alkhami et al. [38] adjusted the analysis for a comprehensive set of factors, including markers of inflammations and DM treatments. Boersma et al.’s multivariable analysis also includes traditional risk factors; however, it underlines the independent predictive value of SAF even after adjusting for these factors. Hence, these findings provide further evidence to support the role of SAF in clinical practice.

Investigating skin AGEs as measured by SAF in the context of macroangiopathy, Aoki et al. [39] aimed to determine whether SAF could surpass IMT and PWV as conventional markers of atherosclerosis. SAF, IMT, and PWV were influenced by age, DM duration, and estimated glomerular filtration rate (eGFR), as well as significantly interrelated. No association was found with coronary and peripheral arterial vasculature damage; however, it was associated with baseline and new stroke occurrences. This finding suggests that SAF could be valuable in non-invasively identifying T2DM patients at increased risk of cerebrovascular events.

Other researchers, such as Kawamoto et al. [46], examined SAF in relation to clinical outcomes in patients who underwent percutaneous coronary interventions. SAF levels were linked with all-cause death, any MI, any stroke, and revascularization, indicating SAF as an independent predictor of these outcomes, irrespective of DM presence [46]. This suggests SAF’s extended utility in CV risk management for this specific group. Additionally, in contrast to the results of Aoki et al. [39], Conway et al. [47] demonstrated a strong connection between SAF and coronary artery calcification (CAC) occurrence and progression in type 1 DM (T1DM) patients. Patients included in this study had a long-standing DM duration, of more than 30 years, thus further broadening SAF’s applicability in CV risk evaluation of different populations exposed to hyperglycemia.

Approaching this topic from slightly different angles and populations, Planas et al. [41] and Jin et al. [40] confirmed the above findings, with regard to the association with CVEs: each study demonstrated that higher SAF levels are associated with an increased risk of CVEs, reinforcing the potential of SAF as a tool for early identification of high-risk patients. Jin et al. [40] focused their research on the direct relationship between SAF levels and CVEs. In contrast, Planas et al. [41] examined how diabetic retinopathy along with SAF would constitute biomarkers for CVEs, suggesting a composite approach to risk assessment, and offering a more detailed risk profiling.

AGEs are recognized as a pathophysiological factor that, along with oxidative stress and inflammation, contribute to the vascular complications of DM. Additionally, microRNA plays a role in the vasculopathy associated with DM. Factors such as insulin resistance, dyslipidemia, and obesity, combined with genetic predispositions, contribute to the development of DM and HBP [48]. These conditions interact bidirectionally, leading to atherosclerosis, endothelial dysfunction, vascular inflammation, and vascular fibrosis, culminating in arterial remodeling. This process ultimately results in both macrovascular and microvascular complications, contributing to CVD and worse outcomes [49].

SAF’s ability to predict both microvascular and macrovascular complications, linking them to prolonged hyperglycemia was also underlined by Wang et al. [43]. They demonstrated SAF as a predictor for various T2DM complications, serving as an independent marker for conditions such as diabetic retinopathy, diabetic kidney disease, CVD, and diabetic neuropathy.

The Rigalleau et al. [44] study similarly explored the relationship between SAF, renal disease, and macroangiopathy in 418 long-standing T2DM patients. SAF was linked to age, renal insufficiency, and smoking and independently associated with macroangiopathy despite chronic kidney disease (CKD) status. Higher SAF values were observed in CKD patients, with SAF inversely related to eGFR, suggesting its potential as a marker for renal function and macrovascular complications in T2DM.

This aligns with existing data, showing that SAF levels are notably higher in T2DM patients, especially those with macrovascular complications. These findings imply that cardiac conditions, patient demographics, types of DM, and the duration of DM could influence the utility of SAF as a marker. This highlights the necessity for further studies to elucidate the most effective application of SAF in managing CV risk, underscoring the complexity of its role across various clinical scenarios.

Several factors identified in the literature can also influence SAF value in patients with CV risk, which may be indirectly associated with CV outcome. These include the patient’s lifestyle, biological markers, aging, and chronic diseases, including T1DM, depression, and DM, associated with complications such as erectile dysfunction or macrovascular complications such as atherosclerotic CVD. Subsequently, we made a thorough presentation of the most relevant aspects of the clinical practice.

### 4.2. Lifestyle Inferences and SAF

During endogenous lipid peroxidation and glycolysis, AGEs and dicarbonyl are produced and are essential elements in the pathophysiology of age-related diseases such as T2DM and CVD. Dicarbonyl sources are food, mainly processed food that includes thermal heating in the Maillard caramelization reaction, such as sugar-rich products—honey, dried fruits, or soft drinks, and also in fermented products, such as soy sauce or balsamic vinegar. It is still unknown to what extent dietary dicarbonyls contribute to circulating dicarbonyls and AGEs, but they are important to remember for a better image of the phenomena. The major dicarbonyls are methylglyoxal (MGO), glyoxal (GO), and 3-deoxyglucosone (3-DG) in >200 commonly consumed foods and drinks. In a study by Maasen et al. [50], the primary dietary source for MGO was coffee, while for GO and 3-DG, it was bread; only dietary MGO and GO were positively associated with their corresponding concentrations in plasma; and dietary MGO, but not GO or 3-DG, were associated with SAF.

Mediterranean diet (MedDiet) and physical activity lower CV risk and mortality. Sánchez et al. [51] evaluated if AGEs are one of the underlying mechanisms to explain this relationship in patients with CVD and T2DM. They observed a negative correlation between adherence to the MedDiet and SAF measurements but not with the level of physical activity. From dietary intake, this was linked to reduced consumption of vegetables, fruits, and nuts and avoidance of sugar-sweetened soft beverages. Moreover, between MedDiet and physical activity, there was no observed interaction in reducing SAF measurements.

Other elements linked to SAF are current smoking and pack-years of smoking but no coffee consumption, as reported in a study by van Waateringe et al. [52]. On the other hand, Eny et al. [53] reported that caffeine contributes to the interindividual variability of SAF values in T1DM due to its metabolites rather than other constituents of coffee. Moreover, caffeine may predict the risk of CVD, prediabetes, and T2DM.

It should be noted that lifestyle can influence SAF values, and through SAF values, it can impact CV risk. By managing dietary intake and promoting a healthy lifestyle, SAF levels can decrease along with CV risk; however, this is only one of the involved mechanisms.

Understanding the significance of AGEs, dicarbonyls, and lifestyle variables in the development of T2DM and CVD emphasizes the crucial necessity of complete dietary and lifestyle management in clinical practice. A comprehensive approach to patient management is required, which involves tailored nutritional recommendations, lifestyle adjustments, and a deep understanding of the molecular pathways that drive illness progression.

### 4.3. Biologic Biomarkers Associations

Hitsumoto et al. [54] report that SAF can be considered novel CV risk factors, alongside in vivo oxidative stress, and high arterial reflection, because they are closely associated with high concentrations of blood hs-cTnT in patients with T2DM. Moreover, in a study by Yoshioka [55], SAF can predict CV risk in patients with T2DM, being correlated with elevated hs-cTnT and NT-proBNP. Another CV risk identified by Hitsumoto [56] is the prediction of the risk of first HF hospitalization in patients with HFpEF.

### 4.4. Ageing and Chronic Diseases Prediction

Reynaert et al. [57] report for aging that AGE products accumulate in arterial walls, endothelium, pulmonary cells, skin collagens, pancreatic β cells, neural tissue, lens proteins, ovaries, skeletal muscles, cardiac myocytes, osteoblasts, chondrocytes, and osteoclasts or as plasmatic levels. Moreover, they are linked with inflammation and chronic diseases such as chronic obstructive pulmonary disease, both T1DM and T2DM, CVD, and osteoporosis. Mooldijk et al. [14] also discovered that SAF is associated with an increased risk of dementia, including Alzheimer’s disease, with this association being stronger among APOE ε4 allele carriers and individuals with DM. Additionally, higher SAF levels correlated with reduced total brain volume, gray matter volume, hippocampal volume, and the presence of lacunes and cerebral microbleeds, indicating AGEs’ potential role in dementia’s pathophysiology.

CKD is another disease where SAF measures AGE because SAF is elevated in patients with mild to moderate forms of disease and is also linked to subclinical atheromatous disease and independently associated with eGFR as reported by Sánchez et al. [58]. Also, Foussard et al. [59] reported that SAF predicts new cancers in T2DM, probably due to AGEs accumulation in tissues, but can be prevented by lifestyle changes such as healthy diet and smoking cessation, alongside with nephroprotection and glucose control. Jin et al. [60] also report that SAF is independently associated with CKD progression.

### 4.5. T1DM

In coeliac disease, Bakker et al. [61] reported no differences compared to controls but showed high SAF levels in T1DM patients compared to controls. Also, in T1DM, SAF is a predictor for macrovascular CV events and eGFR impairment independent of risk factors, as reported by Velayoudom-Cephise et al. [62]. In T1DM, Blanc-Bisson et al. [63] reported that SAF seems able to predict MACE, but further work is required to obtain results on longer-term follow-ups and more MACE to be encountered. Tomaszewski et al. [64] support the existing evidence, reporting that AGEs, as measured by SAF, play a role in aging in T1DM and may also be used as CV markers, inclusively evaluating the risk of all-cause mortality. However, due to small sampling, future research is needed.

Osawa et al. [65] reported an association between SAF and several markers of DM complications and an independent risk factor for IMT even after adjusting for the other established risk factors for atherosclerosis, suggesting its probable predictive role for developing and progressing DM macroangiopathy in T1DM patients. Also, Llauradó et al. [66] reported that SAF is increased in T1DM patients without CVD. However, CVD is associated with arterial stiffness independently of classical glycemic control, disease duration, low-grade inflammation, or CV risk factors, so it can be a valuable tool for predicting arterial stiffness in T1DM.

### 4.6. Depression

Eriksson et al. [67] reported that higher AGE levels, as evaluated by SAF, are present in males as well as in individuals with melancholic depressive symptoms but without a direct causality. Moreover, Spauwen et al. [68] reported in T2DM patients significant and inverse associations between AGEs, evaluated by SAF, and memory, only when it was not adjusted for vascular risk factors and depression. This suggests that AGEs are part of the development of cognitive decline, especially in the decline of memory, seeming that vascular risk factors play an essential role.

### 4.7. Prediction of DM

Prediction of different diseases tends to be evaluated through various risk scores, such as the one reported by Boersma et al. [35]. They showed that a simple model that includes age class, BMI class, and number of parents with DM alongside SAF measurement is like the FINDRISC model in detecting DM at initial screening and during four four-year follow-ups.

Van Waateringe et al. [69] identified SAF as a predictive tool for incident T2DM, CVD, and mortality in the general population, independent of conventional risk factors such as metabolic syndrome (MetS), series glucose level, or HbA1c.

In patients with (MetS), van Waateringe et al. [70] reported that SAF levels are positively associated with the number of individual MetS components and with a higher prevalence of the MetS individual components, leading to the thesis that the accumulation of AGEs may contribute to the pathophysiological development of several CV risk factors.

Smit et al. [71] reported SAF in a decision tree, the performance of DM and IGT diagnostics was similar or superior to conventional risk predictors in at least intermediate risk groups. This follows previous data about SAF’s ability to predict complications of DM. Also, in another paper, Smit et al. [28] reported SAF to be a simple, non-invasive tool for the evaluation of AGE levels and also in identifying patients with, or at high risk for developing, DM, those at high CV risk, including vascular complications.

### 4.8. Erectile Dysfunction

DM has various micro- and macro-vascular complications, including erectile dysfunction (ED). In a study by Kouidrat et al. [72], they reported that SAF as an AGE level marker strongly correlates with the presence and severity of ED in males with DM.

### 4.9. Atherosclerotic CVD

Chen et al. [73] reported the association between AGEs measured by SAF and several endophenotypes of CVD, emphasizing the possibility of the former involvement in processes such as atherosclerosis and arterial stiffness but not in HBP. So, beyond traditional risk factors, SAF may be a marker of vascular aging and subclinical CV changes in male patients with DM or CKD. In another study on Korean patients with T2DM, Choi et al. [74] report that AGEs are linked with PWV and vein age as markers of arterial stiffness and subsequently of CV risk factors as age, weight, or DM duration, fasting blood glucose and proteinuria, being helpful in clinical diagnosis of CVD in T2DM patients. Planas et al. [75] report similar results for SAF and atherosclerotic CVD, respectively, being an independent predictor of coronary artery calcium score (CACs) ≥ 400 in the T2DM population. Van Eupen et al. [76] data support the hypothesis that AGE accumulation, as evaluated by SAF levels, is responsible for the arterial stiffening process and with carotid-femoral PWV, especially in patients with T2DM.

Ying et al. [77] evaluated AGEs evaluated by SAF with an index that includes patients’ age, respectively, AGEage that was measured as AGEs × age/100. This index is correlated with carotid atherosclerosis level, representing a predictor for future CV events in patients with T2DM and being a promising triage option for patients at high risk of CVDs. Another study by Ying et al. [78] showed that the AGEage index is associated with the prevalence of lower extremity arterial disease in T2DM patients independent of HbA1c.

The general limitations across the analyzed studies comprise the type of study, as they vary in study design (retrospective versus longitudinal) and this aspect may affect both the consistency and the comparability of the results. The longitudinal studies performed, such as the ones by Alkhami et al. [38] and Boersma et al. [42], provide stronger evidence of causality; however, limitations are related both to the duration of the follow-up and the participant dropout rate. Other studies may have specific population characteristics [40], or relatively small groups included in the analysis [41], influencing the extrapolation to other or broader populations. Variations in measurement methods, timing, and calibrations needed by the device could also introduce variability in the results [39,43].

In these studies, the impact of skin pigmentation was not mentioned as being consistently addressed, an important bias when ethnically diverse populations are included [39,41,42].

Some other possible sources of bias may include the research settings (such as hospital-based [38] versus community-based [39]), as well as the inclusion criteria, which could potentially introduce selection biases. Confounding factors such as unmeasured or inadequately measured variables (dietary patterns, socioeconomic conditions) could potentially influence the observed associations.

SAF was consistently associated with CV outcomes across studies with a broad range of populations and settings and its potential as a non-invasive CV risk marker in patients with T2DM seems well supported within this context. The studies analyzed, however, report varying strengths in association, and predictive value in different populations might imply SAF integrations into risk models alongside traditional risk factors, instead of replacing them.

Nevertheless, the findings highlight the importance of considering AGE accumulation in the management of T2DM patients. However, the clinical utility of SAF measurement in routine practice requires further validation, considering the limitations and potential sources of bias identified in these studies.

### 4.10. Future Perspectives

Considering the findings, future research and clinical practice could benefit from integrating non-invasive measurements in screening programs. Specifically, using SAF as a screening tool offers a promising approach for early detection and management of high-risk CVD and T2DM. Furthermore, SAF’s potential as a reliable risk estimator for patients with DM and ED highlights its utility in pre-emptively identifying individuals at elevated risk for complications. Additionally, in scenarios requiring immediate CACS evaluation, SAF could serve as an expedient method for identifying high CV-risk populations and optimizing clinical decision-making and patient care.

In order to gain a better understanding of the impact of AGEs on CV risk among patients with T2DM multicentric and ethnically diverse cohorts, standardized measurement protocols for SAF, and comprehensive adjustment for potential confounders are needed.

## 5. Conclusions

In conclusion, SAF is a valuable and non-invasive marker for evaluating CV risk in patients with T2DM. It has promising implications for individualized healthcare and is known for its reliability in associating with CVD and CVEs in the T2DM population. Additionally, SAF measurements have been connected to various complications of diabetes, including retinopathy, nephropathy, and neuropathy. Future studies should focus on unifying outcome metrics, investigating SAF’s predictive accuracy for different types of CVDs, and assessing its effectiveness across various patient populations and clinical scenarios to optimize its contribution to patient care outcomes.

## Figures and Tables

**Table 1:** Newcastle-Ottawa Scale analysis of the included articles.

Author (Reference)	Selection				Comparability	Outcome			Total Score	Quality
	Representativeness of the Exposed Cohort	Selection of the Non-Exposed Cohort	Ascertainment of Exposure	Demonstration that Outcome of Interest Was Not Present at Start of Study	Comparability of Cohorts Based on the Design or Analysis	Assessment of Outcome	Was Follow-Up Long Enough for Outcomes to Occur	Adequacy of Follow-Up of Cohorts		
Alkhami et al. [38], 2024	*	-	*	*	-	*	*	*	6	Good
Aoki et al. [39], 2022	-	*	*	*	-	*	*	*	6	Good
Jin et al. [40], 2021	-	*	*	*	-	*	*	*	6	Good
Planas et al. [41], 2021	-	*	*	*	*	*	*	*	7	good
Boersma et al. [42], 2021	-	*	*	*	-	*	*	*	6	good
Wang et al. [43], 2021	-	*	*	*	-	*	*	*	6	good
Rigalleau et al. [44], 2014	-	*	*	*	-	*	*	*	6	good

“*” indicates that the article meets the criteria mentioned above; “-” indicates that the article does not meet the abovementioned criteria.

**Table 2:** Characteristics of the included studies.

First Author, Publication Year	Country	Study Period	Study Design	Sample Size	Mean Age	SAF Model	Baseline SAF Level	CV Parameters	Outcome of CV Parameters	*p* Value
Alkhami et al. [38], 2024	France	54 months	Cohort	504	62 ± 9	AGE Reader (Diagnoptics, Groningen, The Netherland)	2.68 ± 0.64	CVE	69 (13.69%)	NR
Aoki et al. [39], 2022	Japan	2 years	cross-sectional	845	67 ± 10	AGE Reader (Selista Inc. Tokyo)	158 ± 82	Baseline PAD	χ2 = 6.7	*p* = 0.0096
								Baseline stroke	χ2 = 14.3	*p* < 0.0001
								New CAD	χ2 = 0.3	*p* > 0.05
								New stroke	χ2 = 10.6	*p* = 0.0011
Jin et al. [40], 2021	Hong Kong	45 months	Cohort	3806	60.4 ± 10.4	AGE Reader (DiagnOptics Technologies BV, Groningen, The Netherlands)	2.9 ± 0.6	CVE	172 (4.5%)	*p* < 0.0001
								CHD	73 (2%)	*p* < 0.0001
								Stroke	54 (1.4%)	*p* < 0.0001
								PVD	16 (0.4%)	*p* < 0.0001
								CHF	37 (1.0%)	*p* < 0.0001
Planas et al. [41], 2021	Spain	4.35 years	case-control	187	65.63 ± 6.52	AGE ReaderTM (DiagnOptics TechnologiesBV, Groningen, The Netherlands)	2.68 ± 0.65	CVE	23 (12.3%), 28.2 per 1000 person-years	
Boersma et al. [42], 2021	Netherlands	7 years	Cross-sectional	2349	NR	AGE Reader (DiagnOptics Technologies, Groningen, The Netherlands)	NR	New CVD event	195 (7.6%)	
Wang et al. [43], 2021	China	52 months	cross-sectional	825	NR	Hefei Institutes of Physical Science, Chinese Academy of Sciences	NR	Diabetic CVD risk score		*p* = 0.037
Rigalleau et al. [44], 2014	France	3 years	cross-sectional	418	61.8 ± 10.3	AGE reader (DiagnOptics BV, Groningen, The Netherlands)	2.53 ± 0.62	HBP	Β = 0.15, 95% CI 0.02–0.27	*p* = 0.02

SAF—skin autofluorescence; CV—cardiovascular; AGE – advanced glycation end-products; CVE—cardiovascular events; NR—not reported; PAD—peripheral artery disease; CAD—coronary artery disease; CHD—coronary heart disease; PVD—peripheral vascular disease; CHF—congestive heart failure; CI—confidence interval; CVD—cardiovascular disease; HBP—high blood pressure.
